# Effect of Shielding Gas Arc Welding Process on Cavitation Resistance of Welded Joints of AlMg4.5Mn Alloy

**DOI:** 10.3390/ma16134781

**Published:** 2023-07-02

**Authors:** Marina Dojčinović, Radica Prokić Cvetković, Aleksandar Sedmak, Olivera Popović, Ivana Cvetković, Dorin Radu

**Affiliations:** 1 Faculty of Technology and Metallurgy, University of Belgrade, Karnegijeva 4, 11000 Belgrade, Serbia; rina@tmf.bg.ac.rs; 2 Faculty of Mechanical Engineering, University of Belgrade, Kraljice Marije 16, 11000 Belgrade, Serbia; rprokic@mas.bg.ac.rs (R.P.C.); asedmak@mas.bg.ac.rs (A.S.); opopovic@mas.bg.ac.rs (O.P.); icvetkovic@mas.bg.ac.rs (I.C.); 3 Faculty of Civil Engineering, Transilvania University of Brașov, Turnului Street 5, 500152 Brașov, Romania

**Keywords:** cavitation rate, weld metal, GTAW, GMAW, aluminum alloys

## Abstract

The effect of the shielding gas arc welding process on the cavitation resistance of the three-component aluminum alloy AlMg4.5Mn and its welded joints was investigated. Welding was performed using the GTAW and GMAW processes in a shielded atmosphere of pure argon. After the welding, metallographic tests were performed, and the hardness distribution in the welded joints was determined. The ultrasonic vibration method was used to evaluate the base metal’s and weld metal’s resistance to cavitation. The change in mass was monitored to determine the cavitation rates. The morphology of the surface damage of the base metal and weld metal due to cavitation was monitored using scanning electron microscopy to explain the effect of the shielding gas arc welding process on their resistance to cavitation.

## 1. Introduction

The need to reduce vehicle weight and emissions and to make fuel savings has led to an increase in the use of lightweight materials, such as aluminum alloys. Among them, AlMg4.5Mn alloy stands out, used in many areas, such as tanks for storage and pipelines for transportation of liquid gases, for pressure vessels in general, in vehicles, and more recently as the main material for the construction of yachts and ships [1]. It is characterized by high strength and good resistance to corrosion and wear, as well as relatively good weldability [1]. For these reasons, the use of this alloy and the demands for its improvement and the improvement of joining and shaping techniques of this alloy are increasing [2].

The most common problems encountered when welding Al–Mg alloys are degradation of the mechanical properties in the heat-affected zone, reduction in corrosion resistance [3,4], appearance of pores as a result of hydrogen absorption from the air, appearance of cracks, primarily hot as a result of phase transformations that occur in the weld metal and the heat-affected zone, and the appearance of inclusions—most often Al_2_O_3_ oxides [5]. Other issues include changes in the mechanical properties, which are often related to grain size. During rapid cooling of the fusion zone, intermetallic phase precipitates, such as Mg_2_Si and Al_6_Mn, can form, increasing the hardness. Further, welding can result in changes in the chemical composition and solidification defects or residual stresses generated during solidification and cooling, which can influence the hardness and susceptibility to fracture. Due to the growth of grains in the HAZ, the strength decreases [6,7,8,9]. The addition of elements such as titanium, strontium, and zirconium reduce the mobility of grain boundaries, making the recovery process impossible. In this way, a finer grain is created, which contributes to the quality of the welded joint.

As the most commonly applied joining technique for Al alloys, the gas tungsten arc welding (GTAW) and gas metal arc welding (GMAW) processes are used. The GTAW process is widely used because of the improved control of the heat input. This control is carried out by the correct selection of welding parameters such as the current capacity, welding speed, dimensions and composition of the electrode, flow rate, and composition of the shielding gas [10,11,12]. In addition to the GTAW process, the GMAW process is also used for welding Al and its alloys; it enables higher welding speeds, a narrower HAZ, excellent cathodic cleaning effect, and welding in all positions [13,14,15]. However, there are still a few problems with shielded gas arc welding in respect to the mechanical properties and sensitivity to cracking, especially when GMAW is used. As an illustration, significant reduction in the fatigue strength was obtained in an analysis of the fatigue properties and stress concentration of a 6005 aluminum alloy GMAW-welded lap joint [16]. It was shown that the fatigue strength of the welded joint was just 27% of the fatigue strength of the base metal, mostly due to increased sensitivity to cracking and the geometry of the welded joint. Sensitivity to cracking was also analyzed for Al–Mg alloys, used in the shipbuilding industry, welded by GMAW and friction stir welding (FSW) [17]. It was shown that cracking started mainly through decohesion at the matrix–precipitate interfaces and that the cracking mechanism was trans-crystalline ductile.

One of the most important problems when welding aluminum and its alloys by GTAW and GMAW, in addition to the degradation of the mechanical properties, is the appearance of pores. Pores are formed as a result of adsorption, diffusion, and dissolution of gases on the surface and inside the hardened weld metal. Pores are mainly created by hydrogen that dissolves in aluminum. Since hydrogen has a small atomic diameter, it easily diffuses through the metal in the solid state, so porosity can be observed even in the heat-affected zone. Unlike steel, the pores in aluminum are distributed, mostly, in the interior of the seam, they can also be found near its junction with the base material, and sometimes they can also be observed in the heat-affected zone [18]. The effect of the shielding gas (Ar, He, or N_2_, individually, or in mixtures) on the porosity in GTAW-made welded joints of AlMg4.5Mn alloy has been investigated by performing a microstructural analysis [19]. It was shown that the best results were obtained with the mixture Ar + 59%He + 0.015%N_2_, whereas addition of N_2_ alone did not make any difference.

A more comprehensive experimental study was performed on the same GTAW-made welded joints, including metallography, tensile, hardness, and toughness testing to establish the effects of the shielding atmosphere on the mechanical properties and fracture mechanics parameters of the weld metal [20]. It was concluded that the shielding gas mixture significantly affects the impact toughness, slightly affects the resistance to crack initiation, and somewhat more affects crack growth due to fatigue. More recently, fractography was also used to explain higher values of weld metal toughness as evaluated at three different temperatures using instrumental Charpy pendulum impact testing to measure both the crack initiation energy and the crack growth energy [21].

In addition to GTAW, welded joints of AlMg4.5Mn alloy made by the gas metal arc welding (GMAW) process, with different gas shielding atmospheres, were investigated in [1,20], to assess the effects of the gas mixture on the tensile strength, hardness, impact, and fracture toughness, as well as the fatigue crack growth parameters. The main conclusion was that an increased helium content improves the toughness and fatigue crack growth parameters, as shown in Table 1 for the fatigue crack growth rate, whereas its effect on other mechanical properties is not significant [1,22].

In addition to conventional mechanical properties and corrosion, for alloys used in water it is also important to analyze the resistance to corrosion and cavitation [23,24,25,26,27,28]. Slow strain rate testing (SSRT) was used to study the stress corrosion cracking (SCC) of 5083 Al alloy in a 3.5 percent NaCl solution after superplastic forming and various heat treatments [23], indicating severe SCC and intergranular fracture. Slow strain rate testing (SSRT) was also used to study the effect of the microstructure on the stress corrosion cracking (SCC) susceptibility of Al–Mg alloy sheet containing 6.8% Mg in cold-rolled and fully annealed conditions [24], indicating high SCC susceptibility as well.

Cavitation is the process of the rapid formation, growth, and implosion of bubbles in a fast-flowing liquid. The implosion causes the formation of shock waves and microjets, which, in contact with the surfaces of the elements in hydro-systems, cause their damage in a short time. In the initial stage, the degree of damage is negligible and this is the incubation period. However, repeated impacts of microjets and shock waves during the cavitation effect leads to material damage [25], causing serious problems in vital components, as described in [26]. Cavitation of Al–Mg alloys is rarely investigated, especially for their welded joints. The correlation between the mechanical properties and cavitation resistance of Al–Mg alloy 5083 was analyzed in [27] to obtain the best combination of heat treatments to apply to cast aluminum products. In ref. [28], the cavitation erosion characteristics of the EN AW-6082 aluminum alloy’s surface remelting by GTAW was analyzed. It was shown that the GTAW-remelted layers had cavitation erosion resistance 5–6 times more than the base metal, due to fine graining and microstructure.

In this paper, the cavitation resistance of the welded joints of the three-component aluminum alloy AlMg4.5Mn was investigated by examining the microstructure, determining the hardness distribution, measuring the mass loss, and by fractographic analysis of damaged surfaces. Tests were performed on samples of the base metal and weld metal extracted from the welded joints, made by GTAW and GMAW processes in a shielding atmosphere of pure argon. In this way, the effect of the welding process itself is assessed, whereas the effect of the shielding gas will be analyzed in future research.

## 2. Materials

For the experimental determination of resistance to cavitation, samples of welded joints of AlMg4.5Mn alloy obtained by the GTAW and GMAW processes were used. The chemical composition and mechanical properties of AlMg4.5Mn alloy are shown in Table 2 and Table 3.

**Figure 1:**
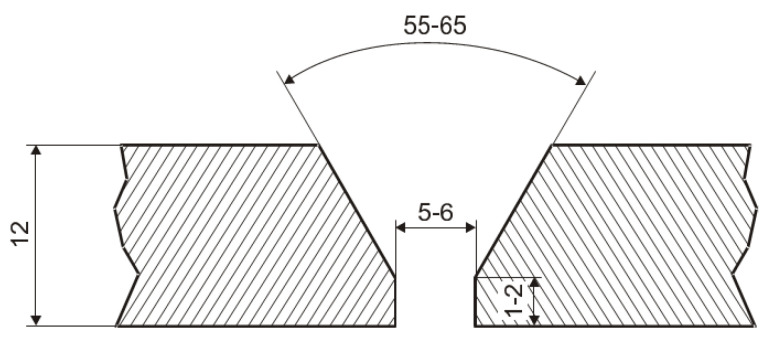
Shape and dimensions of “Y” groove for GMAW.

**Figure 2:**
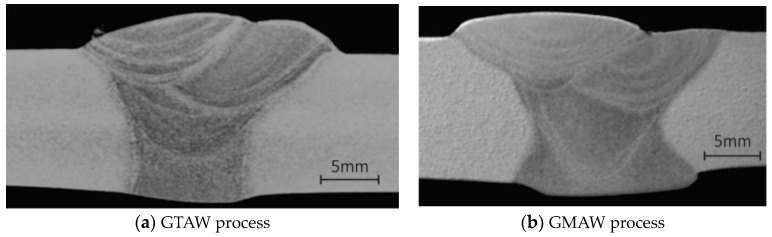
Macrographs of welded joints [17].

Aluminum alloy plates of AlMg4.5Mn, of dimensions 500 mm × 250 mm × 12 mm, were used for welding, and “Y” grooves were made by milling, as shown in Figure 1, for GMAW. The plates were welded in four passes: one root pass and three filling passes. The appearance of macrographs of GTAW- and GMAW-welded joints are shown in Figure 2. The preheating temperature was above 110 °C for GTAW welding and above 70 °C for GMAW welding. Argon 7.0 with a high purity was used as the shielding atmosphere for both the GTAW and GMAW. The gas flow was 17–19 L/min for the GTAW and 15–16 L/min for the GMAW welding. The welding was performed in horizontal position (PA).

For GMAW, welding electrode wire with added zirconium AlMg4.5MnZr in the form of coils weighing 7 kg, Ø1.2 mm was used as a filler metal (Table 4). Zirconium affects the decreasing of the grain size.

For GTAW, welding electrode WZr3 was used and wire made of aluminum alloy AlMg4.5Mn (classifications DIN1732/SG-AlMg4.5Mn or BS2901/5183 or AWS A5.10/ER 5183), Ø5 mm and length 1000 mm, was used as a filler metal (Table 5).

For GTAW, alternative current (AC) was used, whereas direct current reverse polarity (DCEP) was used for the GMAW. The welding parameters, including current, I, voltage, U, welding speed, V_z_, and the resulting heat input, Q = 0.06∙U∙I/V_z_, are shown in Table 6.

## 3. Testing Methods

Before testing the resistance of the samples to the effect of cavitation, metallographic tests were performed to determine the microstructure of the samples. Samples for microstructural tests were cut from the welded plates. These samples were polished and etched in 10% H_3_PO_4_.

In addition, the hardness was measured to determine its distribution in the welded joints, using the standard Vickers method HV5, on the polished samples. The hardness was measured in all zones of the weld joint BM, HAZ, and WM, on 3 levels: along the root, mid-thickness, and close to the surface. The distance between the measuring points was 1.5 mm.

**Figure 3:**
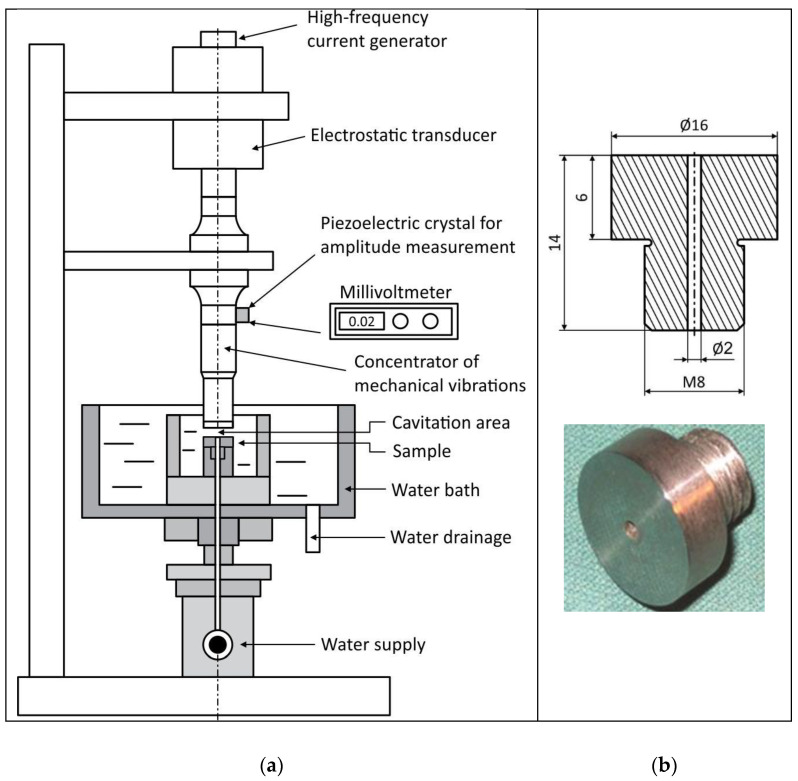
(**a**) Schematic overview of cavitation test setup. (**b**) The test sample.

The ultrasonic vibration method (with a stationary sample), according to the ASTM G32 standard [29], was used to test the cavitation resistance of the AlMg4.5Mn alloy and welded joints. A test sample and the device on which the tests were performed are shown in Figure 3. The ultrasonic vibration method is based on the creation and implosion of cavitation bubbles on the sample’s surface and measuring the mass loss of the sample during the time of exposure to cavitation. The selection of characteristic parameters for testing was performed in accordance with the ASTM G32 standard: frequency of mechanical vibrations was 20 ± 0.2 kHz; amplitude of mechanical vibrations at the top of the concentrator was 50 ± 2 μm; the gap between the test sample and the concentrator was 0.5 mm; water flow was 5–10 mL/s; water bath temperature was 25 ± 1 °C. Before testing, all samples were polished. The change in mass loss was measured with an analytical balance with an accuracy of 0.1 µg. Before measurement, the samples were dried with warm air and kept in a desiccator in order to separate the residual moisture in the material. The samples were measured after 30 min of cavitation exposure for a total test period of 180 min. The results are shown in the mass loss versus time duration exposure diagram. The least squares method was used to construct straight lines whose slope specifies the cavitation rate.

Scanning electron microscopy (SEM) was used to analyze the morphology of the surface damage of the samples. The FESEM Mira Tescan x3m microscope (Brno, The Czech Republic) was used. The analysis of damage to the sample surface was performed after 60, 120, and 180 min of exposure of the samples to the effects of cavitation.

## 4. Results and Discussion

### 4.1. Metallographic Tests

**Figure 4:**
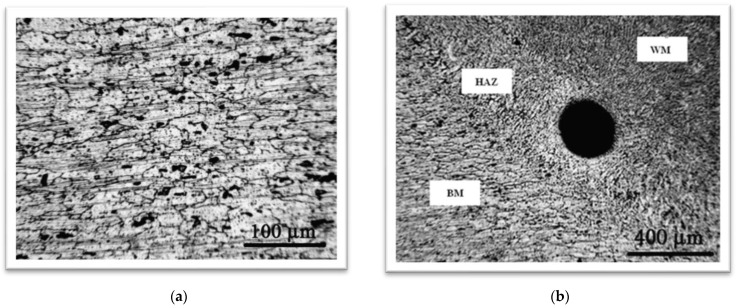
(**a**) The microstructure of aluminum alloy AlMg4.5Mn [21]. (**b**) Large pore in the HAZ of alloy AlMg4.5Mn [19].

**Figure 5:**
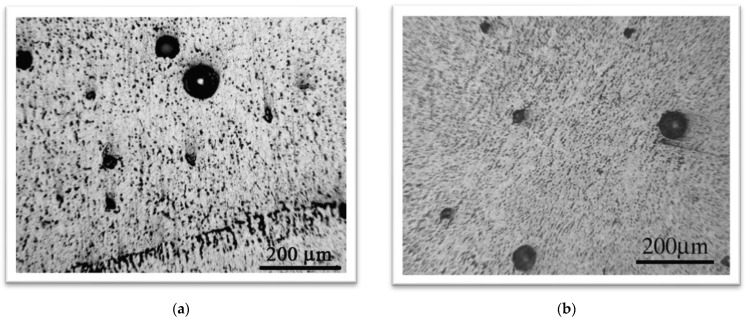
Microstructure of the weld metal: (**a**) GTAW [19], (**b**) GMAW [22].

Figure 4a shows the microstructure of aluminum alloy AlMg4.5Mn. The microstructure consists of metal grains of directional orientation, i.e., it is a typical rolling microstructure with a fine Mg_2_Al_3_ mesh at the grain boundaries. Large dark microconstituents were also observed, which are mostly Mg_2_Si and (Fe, Mn)Al_6_, as explained in [21]. The pores in aluminum alloys are largely distributed within the weld metal, and sometimes in the HAZ, as shown in Figure 4b, where a pore has a large diameter, almost equal to the width of the HAZ [19]. Figure 5 shows the microstructures of the weld metals obtained by the GTAW (Figure 5a) and GMAW (Figure 5b) processes.

The weld metal in both welding processes has a dendritic structure. Intermetallic phases of the type (Fe, Mn, Cr)Al_6_, (Fe, Mn, Cr)SiAl_12_, and Mg_2_Al_3_ are homogeneously distributed in the weld metal. The porosity is dominantly distributed around the interface between two passes and the interface between the base metal and the weld metal. A somewhat lower porosity and a finer-grained microstructure is obtained with the GMAW process [22]. This is probably the result of the use of a filler material with the small addition of zirconium. It has been found that zirconium may be used to promote a fine grain size, acting as nuclei for the formation of very fine grains during solidification. The grain size has a dominating effect on the mechanical properties in the case of GMAW, while the shielding gas composition effect is dominant for GTAW.

### 4.2. Hardness Test

The results for the hardness measurements are shown in Table 7. The hardness of the base material ranged from 67.3–74.4 HV. The hardness of the weld metal produced by the GTAW welding process ranged from 67.1–78.5 HV, while that of the weld metal produced by the GMAW process was 70–88.5 HV. The somewhat higher values of the hardness of the weld metal obtained by the GMAW process are the result of a finer grain structure. The hardness of the HAZ was 67.5–76.6 in the GTAW-welded joint, and 71.3–81.5 in the GMAW-welded joint. The hardness values indicate a homogeneous microstructure since the values are in the range of ±7.5%, except in the case of the BM made by GMAW where the range was ±12%. This appears to be the consequence of a harder surface layer obtained by the GMAW process. One can see that the hardness is highest in WM made by GMAW and lowest in BM, with WM made by GTAW closer to BM than to WM made by GMAW. The hardness in the HAZ follows the same trend.

### 4.3. Cavitation Rates

**Figure 6:**
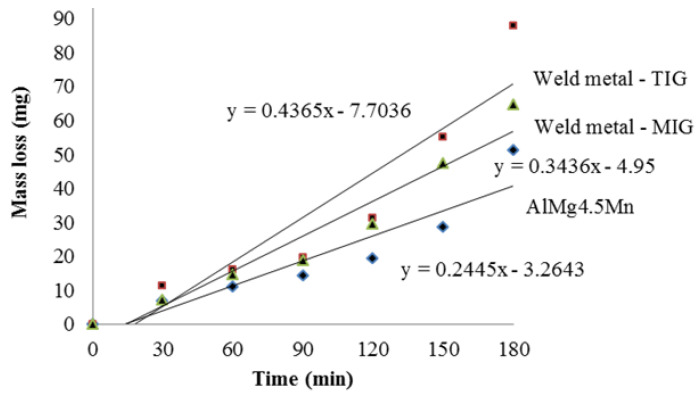
Cumulative mass loss during total exposure time.

The results of measuring the mass loss of the samples (base material AlMg4.5Mn alloy, GTAW weld metal, GMAW weld metal) under the effect of cavitation during the testing time are shown in Figure 6. The mass loss caused by cavitation damage is plotted on the ordinate and the time intervals are given on the abscissa. Using the least squares method, the points of the diagram are approximated by a straight line with slope proportional to the mass loss after the total time of cavitation, which defines the cavitation rate. Therefore, an increase in the slope indicates a reduction in cavitation resistance. The intersection point of the straight line and the abscissa indicates the incubation period, i.e., the period of time in which there is no loss in material mass under the effect of cavitation. The incubation period of all of the samples was about 10 min (Figure 6). In this period, there was no separation of material particles under the effect of cavitation during the test.

Table 8 shows the mass losses of all samples after the cavitation test, which lasted 180 min. The table shows the calculated cavitation rates of all samples. The base material of the AlMg4.5Mn alloy has the highest resistance to the effect of cavitation (the lowest total mass loss and the lowest cavitation rate). The weld metal obtained by the GMAW process has a higher resistance to the effect of cavitation than the weld metal obtained by the GTAW process. This behavior is due to the smaller porosity and finer microstructure of the GMAW WM process. One should note that there is no correlation with hardness (the highest in GMAW WM, lowest in BM and close to that in GTAW WM) since the cavitation rate is the highest in GTAW WM, lowest in BM, and in between in GMAW WM.

### 4.4. Morphology of Damaged Surfaces

**Figure 7:**
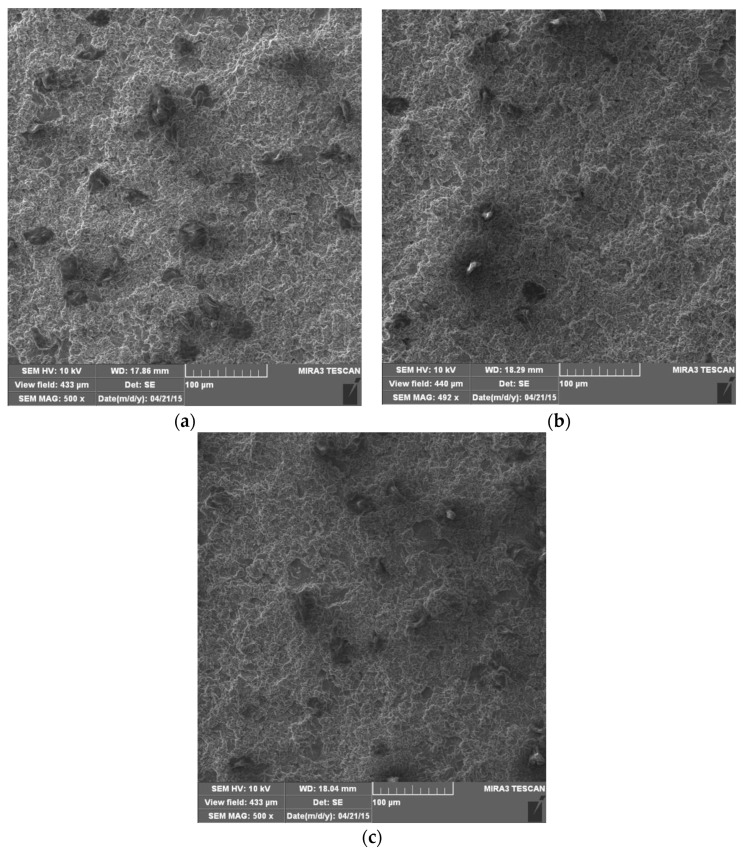
SEM microphotographs of damaged surfaces after 60 min: (**a**) base metal; (**b**) weld metal, GTAW; (**c**) weld metal, GMAW.

Scanning electron microscopy (SEM) was used to analyze the surface damage of all of the tested samples. Figure 7 shows microphotographs of the surfaces after 60 min of exposure to the effect of cavitation. In all samples, the presence of a uniform undulation of the surface exposed to the effect of cavitation was observed. Small pits appear, but in a matrix that is soft. There is still a pronounced grain boundary network because it forms Mg_2_Al_3_ which is a hard phase. Dark microconstituents are also present, especially on the surface of the base material sample (Figure 7a), which were observed on the surface of the samples before testing.

**Figure 8:**
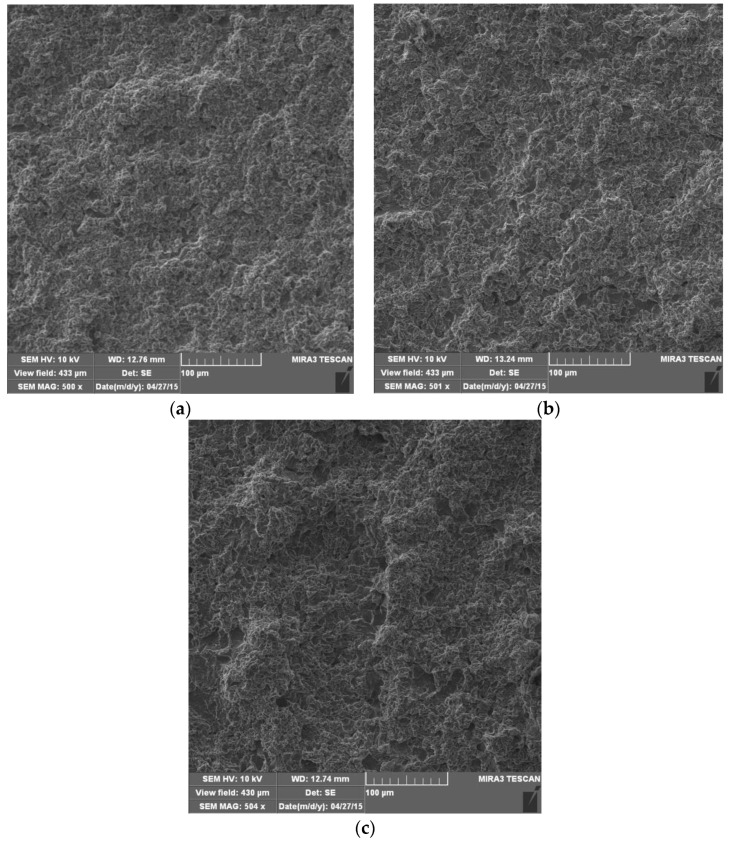
SEM microphotographs of damaged surfaces after 120 min: (**a**) base metal; (**b**) weld metal, GTAW; (**c**) weld metal, GMAW.

After 120 min of cavitation action, a greater degree of plastic deformation than in the previous test interval was observed in all samples (Figure 8), which is reflected in more expressed surface undulation. Pits created by the removal of microconstituents were observed. The degree of base damage is significantly higher than in the previous test interval, which is in accordance with the mass loss measurements (Figure 6).

**Figure 9:**
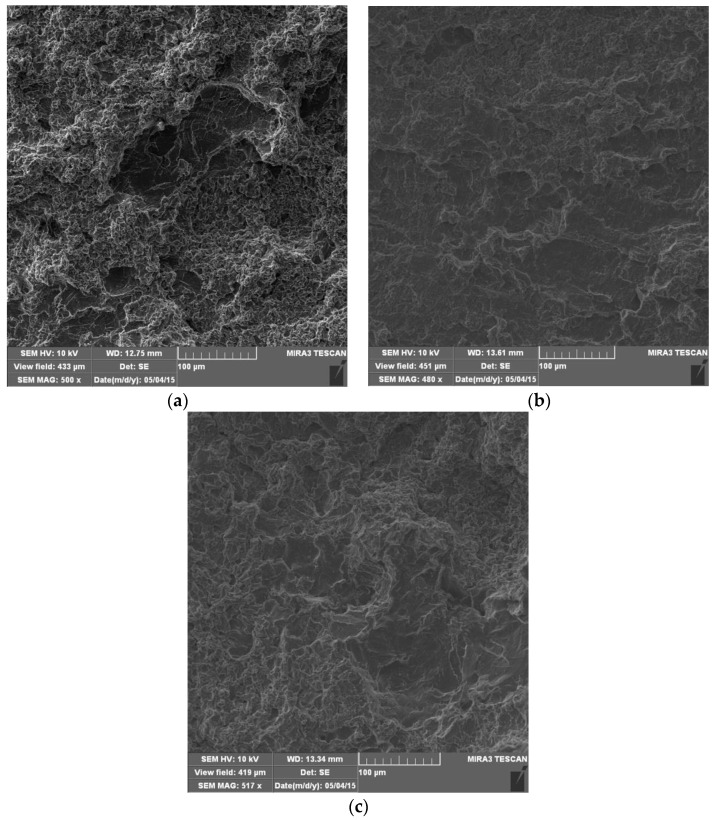
SEM microphotographs of damaged surfaces after 180 min: (**a**) base metal; (**b**) weld metal GTAW; (**c**) weld metal, GMAW.

**Figure 10:**
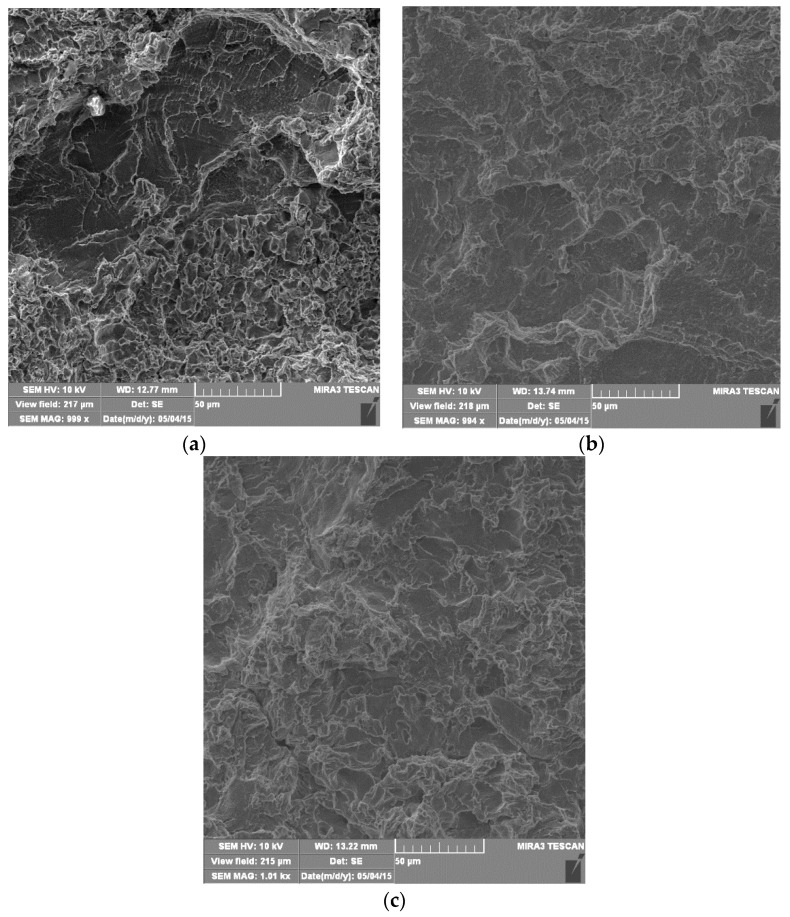
SEM microphotographs of craters with fatigue-like striations after 180 min: (**a**) base metal; (**b**) weld metal, GTAW; (**c**) weld metal, GMAW.

After 180 min of testing, it was observed that a large amount of material had been removed in all of the tested samples. That is why priority damaged areas cannot be identified (Figure 9). The surfaces appear distinctly wrinkled, with ridges of irregular shapes, with a morphology reminiscent of a ductile fracture mechanism. Surface damage is so much expressed that pits are not visible because they have merged and formed craters. The appearance of a fatigue-like damage mechanism was observed in all samples, as shown in detail in Figure 10. Craters with pronounced circumferential striations are clearly visible, which is a typical form of fatigue damage. The craters are most expressed in BM (Figure 9a), due to the fatigue damage mechanism, being in accordance with its highest cavitation resistance.

## 5. Conclusions

The cavitation resistance of the welded joints of aluminum alloy AlMg4.5Mn obtained by the GTAW and GMAW processes, as well as the base metal, was investigated by examining the microstructure, determining the hardness distribution, measuring the mass loss during the cavitation process, and fractographic examination of the damaged surface.

The microstructure was finer and with less pores in the case of GMAW, as a consequence of using a filler metal with Zr, which creates nuclei for the formation of very fine grains during solidification. One should note that GTAW can produce higher quality welded joints with less pores if shielding gas mixtures are used, but here the focus was on a comparison with GMAW with pure Ar as the shielding gas.

The distribution of hardness in the welded joints was determined and the results showed slightly higher hardness values of the weld metal obtained by the GMAW process compared to the GTAW process, which is a consequence of the finer-grained microstructure of the weld metal. However, hardness is not an important factor here, since it cannot be correlated with the cavitation rates for BM and MW made by GMAW and GTAW.

The base material, AlMg4.5Mn alloy, has the highest resistance to the effect of cavitation (the lowest total mass loss and the lowest value of the cavitation rate). The weld metal obtained by the GMAW process has a higher resistance to the effect of cavitation than the weld metal obtained by the GTAW process. This behavior is a consequence of the lower porosity and finer microstructure of the weld metal obtained by the GMAW process.

Finally, one can conclude from the morphology of the samples’ surface damage during the cavitation that all tested samples showed fatigue characteristics. Fatigue behavior should be investigated as the next step to provide a better insight into its effects on cavitation resistance.

The composition of the shielding gas is another very important issue to be investigated in the future, since its effect on the weld metal properties, including cavitation resistance, is expected to be significant.

## Figures and Tables

**Table 1:** Data for fatigue crack growth using different shielding gases.

Shielding Gas	ΔK_th_, MPa∙m^1/2^	Coefficient C	Coefficient m	da/dN, m/cyc ΔK = 7 MPa∙m^1/2^
Ar	2.88	4.44 × 10^−10^	3.86	6.87 × 10^−7^
Ar + 0.0307%O_2_	2.83	4.17 × 10^−10^	3.81	8.12 × 10^−7^
Ar + 48%He + 0.0290%O_2_	3.02	1.05 × 10^−10^	4.07	2.89 × 10^−7^

**Table 2:** Chemical composition of aluminum alloy AlMg4.5Mn.

Element	Si	Fe	Cu	Mn	Mg	Zn	Cr	Ti
mass %	0.13	0.21	0.04	0.66	3.95	0.03	0.06	0.025

**Table 3:** Mechanical properties of aluminum alloy AlMg4.5Mn.

	Tensile Strength R_m_ (MPa)	Yield Strength R_0.2_ (MPa)	Elongation A (%)	Total Impact Energy (J)
Rolling direction	293	133	25	41
Transverse direction	304	143	24	32

**Table 4:** Chemical composition of filler material (GMAW process, remaining is Al).

Element	Si	Fe	Cu	Mn	Mg	Zn	Cr	Ti	Zr
mass %	0.07	0.21	0.01	0.71	4.6	0.02	0.07	0.09	0.11

**Table 5:** Chemical composition of filler metal (GTAW process, remaining is Al).

Element	Si	Fe	Cu	Mn	Mg	Zn	Cr	Ti	Others	
									Each	Total
mass %	<0.40	<0.40	<0.10	0.5–1.0	4.3–5.2	<0.25	0.05–0.25	0.15	<0.05	<0.15

**Table 6:** Welding parameters.

Process	I (A)	U (V)	V_z_ (cm/min)	Q (kJ/cm)
GTAW	215–220	20–22	11–15	19.4–23.5
GMAW	160–170	20–22	27–32	7.0–7.1

**Table 7:** Hardness measurement, HV_5._

	GTAW			GMAW		
	Face	Center	Root	Face	Center	Root
BM	74.0			78.6		
	74.4	67.3	71.3	79.8	72.3	77.9
	72.2	70.1	70.6	80.2	78.4	81.6
	71.6	68.5	73.2	76.2	72.7	83.1
	72.0	68.6	72.0	81.1	74.8	82.1
	73.5	74.9	72.2	80.3	73.9	83.3
HAZ	75.5	76.6	70.9	79.9	78.6	81.5
WM	74.7	67.1	69.3	74.0	78.2	81.7
	73.4	76.3	75.1	73.6	82.2	84.3
	74.6	74.4	77.3	82.6	70.8	84.5
	73.4	76.8	71.1	79.1	79.2	79.1
	74.7	78.5	74.1	75.1	77.4	84.1
	73.3	73.4	76.2	70.0	74.3	81.3
	73.3	75.2	74.8	71.2	75.2	88.5
	73.1	71.4	75.1	76.5	77.2	81.8
HAZ	74.3	67.5	72.3	71.3	74.2	79.4
BM	71.1	68.7	71.1	73.1	70.4	78.8
	71.4	70.7	71.8	76.1	71.3	79.6
	71.7	74.3	69.0	79.4	72.3	82.1
	74.2	72.5	73.8	78.5	78.5	82.6
	73.4			76.8		

**Table 8:** Total mass losses and cavitation rates of the tested samples.

Sample	Total Mass Loss (mg)	Cavitation Rates (mg/min)
Base metal—AlMg4.5Mn	51.3	0.285
Weld metal—GTAW	87.9	0.488
Weld metal—GMAW	64.5	0.358
